# Impact of micro‐ and macrovascular complications of type 2 diabetes on quality of life: Insights from the DISCOVER prospective cohort study

**DOI:** 10.1002/edm2.321

**Published:** 2022-01-14

**Authors:** Suzanne V. Arnold, Kamlesh Khunti, Fengming Tang, Hungta Chen, Antonio Nicolucci, Marilia B. Gomes, Linong Ji, Marina V. Shestakova, Hirotaka Watada, Andrew Cooper, Peter Fenici, Niklas Hammar, Jesús Medina, Mikhail Kosiborod, the DISCOVER investigators

**Affiliations:** ^1^ Saint Luke’s Mid America Heart Institute Kansas City Missouri USA; ^2^ University of Missouri Kansas City Missouri USA; ^3^ University of Leicester Leicester UK; ^4^ AstraZeneca Gaithersburg Maryland USA; ^5^ Center for Outcomes Research and Clinical Epidemiology Pescara Italy; ^6^ Rio de Janeiro State University Rio de Janeiro Brazil; ^7^ Peking University People’s Hospital Beijing China; ^8^ Endocrinology Research Centre Diabetes Institute Moscow Russian Federation; ^9^ Graduate School of Medicine Juntendo University Tokyo Japan; ^10^ AstraZeneca Cambridge UK; ^11^ AstraZeneca Gothenburg Mölndal Sweden; ^12^ Institute of Environmental Medicine Karolinska Institutet Stockholm Sweden; ^13^ AstraZeneca Madrid Spain; ^14^ The George Institute for Global Health and University of New South Wales Sydney Australia

**Keywords:** quality of life, type 2 diabetes, vascular disease

## Abstract

**Background:**

The key goals of management in patients with type 2 diabetes (T2D) are to prolong life and improve quality of life. Micro‐ and macrovascular complications of T2D not only increase the risk of morbidity and mortality, but cross‐sectional studies indicate they may also worsen quality of life. We prospectively examined the association of complications that developed during the follow‐up with concurrent changes in quality of life.

**Materials and methods:**

DISCOVER is a multinational, prospective, observational cohort study of T2D patients enrolled at initiation of second‐line glucose‐lowering therapy. Quality of life was assessed with the SF‐36 Physical (PCS) and Mental Components Summary (MCS) scores at baseline, 6 months, and 1, 2 and 3 years. Hierarchical repeated measures regression models for PCS and MCS were constructed with complications included as time‐dependent covariates; first each complication was modelled alone and then second including all interval complications (to account for different complications occurring in the same patient).

**Results:**

Among 7830 patients with T2D from 30 countries (mean age 56.6 years, 47.6% women, mean duration of T2D 5.6 years), baseline mean SF‐36 PCS was 48.0 ± 7.8 and SF‐36 MCS was 45.5 ± 10.4. At baseline, 1422 (18.2%) patients had a known microvascular complication, and 966 (12.3%) had a macrovascular complication. Over the 3 years of the study, 641 (12.0%) developed a new microvascular complication (most commonly neuropathy) and 372 (5.8%) developed a new macrovascular complication (most commonly coronary disease). New diagnoses of coronary disease, peripheral artery disease, heart failure and neuropathy were each associated with subsequent moderate reductions in SF‐36 PCS (range 0.7 to 1.6 points) and new cerebrovascular disease was associated with a reduction in SF‐36 MCS (2.6 points). Results were consistent when all interval complications were considered in the same model.

**Conclusion:**

In a prospective, multinational study of patients with T2D, the development of macrovascular complications and neuropathy was associated with decreases in both physical and mental quality of life. Our results provide additional support for clinicians to focus on the prevention, detection and management of the complications of T2D.

## Graphical abstract

We used prospective data from 7830 patients with T2D from 30 countries with 3 years of follow‐up to examine the association of development of complications of T2D with changes in quality of life. We found that new diagnoses of coronary disease, cerebrovascular disease, peripheral artery disease, heart failure and neuropathy were each associated with subsequent moderate reductions in both SF‐36 PCS and MCS (range 0.5 to 2.7 points). These findings further highlight prevention of T2D complications as the key to preserving quality of life.

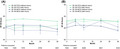

## 1 INTRODUCTION

While preventing morbidity and prolonging life are key goals of treatment in patients with type 2 diabetes (T2D) and are the focus of many studies, improving or maintaining quality of life may be of equal or even greater importance to patients. Several factors have the potential to adversely impact the quality of life of patients with T2D, including feeling challenged by glycaemic control, dietary restrictions, hypoglycaemia, polypharmacy and medication side effects. Another important aspect of living with diabetes is the development of microvascular and macrovascular complications, which are not only common^1^, ^2^ but also increase the risk of additional morbidity^2^, ^3^ and mortality.^4^, ^5^, ^6^ Beyond survival and hospitalization, complications may also adversely impact the patient's quality of life, with cross‐sectional studies indicating that diabetes‐related complications are associated with an increased risk of depression or anxiety,^7^ lower treatment satisfaction scores^8^ and worse quality of life.^9^ Complications in patients with type 1 diabetes have also been associated with lower scores on all subscales of the 36‐item Short‐Form Health Survey (SF‐36), with a similar but weaker association in patients with T2D.^10^, ^11^ Regarding specific complications, neuropathy, cardiovascular disease and end‐stage renal disease have each been found to be associated with decreased scores on the SF‐36.^12^, ^13^

A key limitation in these prior studies is their cross‐sectional design, which has substantial risk of confounding, as patients with particular complications also have other factors that impact quality of life that cannot be fully accounted for with multivariable adjustment. Furthermore, as multiple complications often cluster in the same patients, understanding the true impact of any particular complication requires concurrent adjustment for other complications. As such, we used the DISCOVER prospective global study to examine the association of development of new complications with concurrent changes in quality of life. This longitudinal approach allows each patient to serve as his or her own control, thereby reducing bias due to confounding and more effectively isolating the impact of the new complication on quality of life.

## 2 METHODS

### 2.1 Study protocol

The DISCOVER study is a multinational, prospective, observational study of individuals with T2D being initiated on second‐line glucose‐lowering medication (either add‐on or switching).^14^ Between December 2014 and June 2016, consecutive eligible adults were enrolled from 38 countries (Supplemental Table S1) and followed at 6, 12, 24 and 36 months.^15^ Patients were excluded who were pregnant, undergoing dialysis, had a history of renal transplant or were treated with an injectable agent or herbal remedy/natural medicine alone as a first‐line agent. Patients from China (*n* = 1292) were excluded from this analysis due to regulations on data privacy released during the study. Data from Canada, Denmark, Japan and Norway (*n* = 2255) were excluded, as hospitalization data from these countries were incomplete. Data from Bahrain, Kuwait and Oman (*n* = 152) were excluded, as the SF‐36 was not collected in these countries.

Demographics, comorbidities, interval events, laboratory data and medications were prospectively collected using standardized data collection forms. In line with the observational nature of the study, patients were not obliged to attend study visits (data were recorded at clinical follow‐up visits), and clinical data were measured and recorded according to routine clinical practice at each site. The study protocol was approved by the appropriate clinical research ethics committees in each participating country and by the institutional review board at each site. All participants provided written informed consent. The data that support the findings of this study are available from the corresponding author or study sponsor upon reasonable request.

### 2.2 Complications

Microvascular and macrovascular complications were assessed at baseline and each follow‐up time point using a combination of outpatient visits, emergency room visits and hospitalizations. Diagnoses of complications were not adjudicated and relied on the judgement of the local investigators. Microvascular complications included retinopathy, neuropathy (peripheral, autonomic), erectile dysfunction and nephropathy (albuminuria, chronic kidney disease). Macrovascular complications included coronary artery disease (CAD, myocardial infarction, coronary revascularization, angina), cerebrovascular disease (CVD, stroke, transient ischaemic attack, carotid endarterectomy or stenting), peripheral artery disease (PAD, diabetic foot, amputation) and heart failure. Interval complications were defined as complications that were not diagnosed at the time of patient enrolment but developed (or were recognized) during the prospective follow‐up of the study.

### 2.3 Quality of life assessment

Quality of life was assessed using the SF‐36, which is a generic health status measure that consists of 36 questions across eight domains: physical functioning, role physical, bodily pain, general health, vitality, role emotional, social functioning and mental health.^16^ The SF‐36 also provides a physical component summary (PCS) and mental component summary (MCS), which were the primary outcomes of interest for the current study. Scores for the PCS and MCS are scaled to an overall US population mean of 50 and standard deviation of 10; higher scores indicate better health status, and the minimal clinically important change is ~2.5 points.^17^ Patients with baseline SF‐36 and at least one follow‐up assessment were included.

### 2.4 Statistical analysis

Baseline characteristics of patients who were eligible but missing follow‐up data were compared with those in the analytic cohort using standardized differences (>10% difference is considered clinically relevant). Within the analytic cohort, for descriptive purposes, we compared the characteristics of patients with versus without complications at enrolment, including baseline PCS and MCS scores. To examine the unadjusted association of interval complications with changes in quality of life, we compared the percentage of patients who had a ≥2.5‐point decrease in PCS or MCS from baseline to last available follow‐up between those with versus without each of the interval complications using chi‐square tests.

Our primary analysis involved construction of hierarchical repeated measures linear models for (1) PCS and (2) MCS with each interval complication entered as a time‐dependent covariate and adjusted for baseline PCS or MCS, respectively. These models were first done with each complication separately (ie one model for each complication) and then as a single model with all interval complications included (as patients could experience more than one complication during follow‐up). Patients were considered to serve as their own control, with baseline PCS or MCS included in the models, and thus, no additional adjustment was made for patient factors. All analyses were conducted using SAS version 9.4 (SAS Institute, Cary, North Carolina), with statistical significance determined by *p *< 0.05. As these analyses were considered exploratory, there was no statistical adjustment for multiple testing.

## 3 RESULTS

### 3.1 Patient population

A total of 15,983 people with T2D from 38 countries who were initiating 2nd‐line glucose‐lowering therapy were enrolled in DISCOVER between 2014 and 2016. We excluded 3699 from eight countries due to complete data being unavailable at the time of the analysis for administrative reasons (China), incomplete data on hospitalizations (Canada, Denmark, Japan, Norway) or lack of SF‐36 collection (Bahrain, Kuwait, Oman). We also excluded 50 patients with missing data on baseline complications, 3978 patients with missing baseline SF‐36 scores and 426 additional patients with no follow‐up SF‐36 data. As such, our analytic cohort included 7830 participants from 30 countries. Patients excluded due to missing data on complications during follow‐up or SF‐36 data were more likely to be non‐smokers, had higher body mass indices and higher blood pressure; otherwise were similar to those in the analytic cohort in terms of demographics, laboratory data, comorbidities, medications and baseline complication burden (Supplemental Table S2). Mean age (±SD) of the analytic cohort was 56.6 ± 11.6 years, 47.6% were women, 10.8% were current smokers, mean HbA1c was 8.4 ± 1.7% and mean duration of T2D at time of enrolment was 5.6 ± 5.1 years.

**FIGURE 1:**
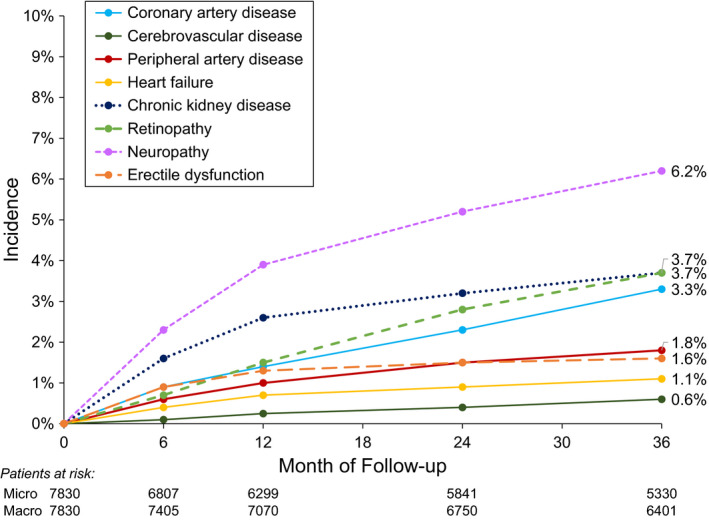
Incidence of complications over 3 years of follow‐up

There were 2076 patients (26.5%) who had a vascular complication at enrolment, and these patients were more likely to be older age, current or former smokers, and have a longer duration of T2D (Table 1). Neuropathy was the most common baseline complication at 9.3%, followed by coronary artery disease in 8.6% and chronic kidney disease in 7.1% of patients (Table 2). There were 1397 participants (17.8%) who developed at least one new microvascular complication over the course of the study (most commonly, neuropathy) and 596 (7.6%) who developed at least one new macrovascular complication (most commonly, coronary artery disease; Figure 1).

**TABLE 1:** Patient characteristics according to T2D complications at enrolment

	Complication *n* = 2076 (26.5%)	No complication *n* = 5754 (73.5%)	Standardized differences	*p*‐value
Age, years	60.7 ± 11.2 (2076)	55.1 ± 11.5 (5754)	49.6%	<0.001
Female sex	877 (42.2%)	2849 (49.5%)	14.6%	<0.001
Tobacco smoking			26.8%	<0.001
Non‐smoker	1368 (67.7%)	4462 (78.8%)		
Former smoker	400 (19.8%)	629 (11.1%)		
Current smoker	253 (12.5%)	575 (10.1%)		
Body mass index, kg/m^2^	30.1 ± 5.7 (1996)	29.4 ± 5.7 (5375)	12.6%	<0.001
Duration of diabetes, years	6.8 ± 5.8 (2057)	5.2 ± 4.8 (5698)	29.9%	<0.001
Systolic blood pressure, mmHg	132.2 ± 16.9 (2017)	131.2 ± 15.7 (5569)	6.1%	0.017
Diastolic blood pressure, mmHg	79.9 ± 9.7 (2017)	79.9 ± 9.2 (5569)	0.0%	0.99
HbA1c, %	8.5 ± 1.7 (1703)	8.4 ± 1.6 (4265)	3.7%	0.192
Total cholesterol, mg/dL	183.3 ± 50.3 (1443)	186.3 ± 46.6 (2945)	6.4%	0.044
Low‐density lipoprotein cholesterol, mg/dL	104.2 ± 40.8 (1229)	109.3 ± 39.6 (2624)	12.8%	<0.001
Triglycerides, mg/dL	185.3 ± 109.9 (1373)	180.9 ± 129.4 (2889)	3.7%	0.278
High‐density lipoprotein cholesterol, mg/dL	44.6 ± 13.9 (1244)	44.7 ± 12.3 (2623)	1.3%	0.705
Creatinine, mg/dL	1.1 ± 0.9 (1472)	1.0 ± 1.1 (2677)	8.1%	0.014
ACE‐I or ARB	1165 (56.1%)	1925 (33.5%)	46.8%	<0.001
Beta blocker	635 (30.6%)	588 (10.2%)	52.2%	<0.001
Statin	1119 (53.9%)	2373 (41.2%)	25.6%	<0.001
Aspirin	718 (34.6%)	621 (10.8%)	59.3%	<0.001

Note

Data are presented as mean ± SD (number of patients with data) or *n* (%).

ACE‐I, angiotensin‐converting enzyme inhibitor; ARB, angiotensin II receptor blocker.

**TABLE 2:** Unadjusted association of baseline complications with baseline quality of life

	*N* (%)	SF‐36 Physical Component Summary			SF‐36 Mental Component Summary		
		Mean (SD)		*p*‐value	Mean (SD)		*p*‐value
		With complication	Without complication		With complication	Without complication	
Any complication	2076 (26.5%)	47.3 (47.0–47.6)	48.3 (48.1–48.5)	<0.001	46.0 (45.6–46.4)	45.3 (45.0–45.6)	0.006
Macrovascular complication	966 (12.3%)	46.3 (45.8–46.8)	48.3 (48.1–48.5)	<0.001	45.3 (44.7–45.9)	45.5 (45.3–45.7)	0.534
Coronary artery disease	677 (8.6%)	46.6 (46.0–47.2)	48.2 (48.0–48.4)	<0.001	45.4 (44.6–46.2)	45.5 (45.3–45.7)	0.824
Cerebrovascular disease	159 (2.0%)	45.6 (44.3–46.9)	48.1 (47.9–48.3)	<0.001	44.8 (43.1–46.5)	45.5 (45.3–45.7)	0.419
Peripheral artery disease	122 (1.6%)	44.9 (43.3–46.5)	48.1 (47.9–48.3)	<0.001	47.3 (45.3–49.3)	45.4 (45.2–45.6)	0.055
Heart failure	314 (4.0%)	45.4 (44.5–46.3)	48.1 (47.9–48.3)	<0.001	43.8 (42.7–44.9)	45.5 (45.3–45.7)	0.003
Microvascular complication	1422 (18.2%)	47.6 (47.2–48.0)	48.1 (47.9–48.3)	0.021	46.5 (46.0–47.0)	45.2 (44.9–45.5)	<0.001
Chronic kidney disease	559 (7.1%)	47.9 (47.2–48.6)	48.0 (47.8–48.2)	0.700	47.9 (47.0–48.8)	45.3 (45.1–45.5)	<0.001
Retinopathy	238 (2.7%)	47.4 (46.3–48.5)	48.0 (47.8–48.2)	0.177	46.2 (44.9–47.5)	45.5 (45.3–45.7)	0.298
Neuropathy	729 (9.3%)	46.8 (46.3–47.3)	48.2 (48.0–48.4)	<0.001	45.3 (44.6–46.0)	45.5 (45.3–45.7)	0.614
Erectile dysfunction	208 (2.7%)	48.3 (47.3–49.3)	48.0 (47.8–48.2)	0.609	45.8 (44.4–47.2)	45.5 (45.3–45.7)	0.612

### 3.2 Quality of life

Mean SF‐36 PCS score at baseline was 48.0 (95% CI 47.8–48.2), and mean SF‐36 MCS was 45.5 (95% CI 45.3–45.7). Mean SF‐36 PCS scores were lower in those with versus without known complications at the time of enrolment (47.3 [95% CI 47.0–47.6] vs. 48.3 [95% CI 48.1–48.5], *p *< 0.001) whereas SF‐36 MCS scores were slightly higher in those with complications (46.0 [95% CI 45.6–46.4] vs. 45.3 [95% CI 45.0–45.6], *p *= 0.006; Table 2). Individually, each of macrovascular complications and neuropathy was associated with lower baseline SF‐36 PCS scores whereas SF‐36 MCS scores were similar in those with and without baseline complications.

**FIGURE 2:**
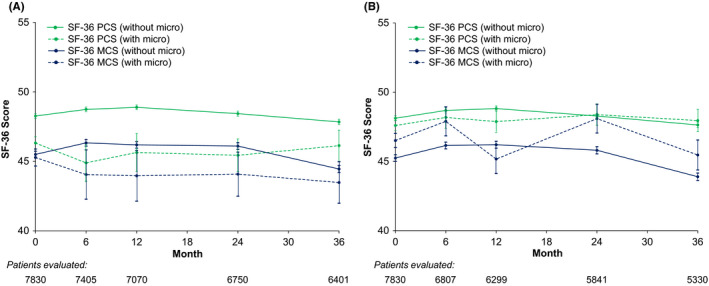
Short‐form 36 physical and mental components summary score over 3 years of follow‐up

Over the 3 years of the study, mean SF‐36 PCS and MCS scores remained roughly stable, with a slight decrease between years 2 and 3 (Figure 2). Those with macrovascular complications had lower SF‐36 PCS and MCS scores at all time points compared with those without macrovascular complications, whereas there was little association between microvascular complications and SF‐36 scores over time. Overall, 34.4% of patients had a clinically meaningful decrease in SF‐36 PCS scores over follow‐up (≥2.5‐point decrease), and 37.7% had a meaningful decrease in SF‐36 MCS scores. Among individual complications, fewer patients with interval coronary artery disease had significant declines in SF‐36 PCS scores compared to patients without coronary disease (26.3% vs. 34.5%, *p *= 0.12; Table 3), and patients with interval cerebrovascular disease were more likely to have significant reductions in SF‐36 MCS scores compared to those with cerebrovascular disease (55.3% vs. 38.3%; Table 4).

**TABLE 3:** Association of interval complications with physical quality of life

	Model estimates				% with ≥2.5‐Point reduction		
	Unadjusted^a^	*p*‐value	Adjusted^b^	*p*‐value	With Complication	Without Complication	*p*‐value
Coronary artery disease	−0.7 (−1.4 to −0.1)	0.031	−0.7 (−1.4 to 0.1)	0.073	26.3%	34.6%	0.012
Cerebrovascular disease	−1.6 (−3.3 to 0.2)	0.077	−1.2 (−3.2 to 0.8)	0.227	42.1%	34.2%	0.307
Peripheral artery disease	−1.5 (−2.4 to −0.6)	0.001	−1.3 (−2.3 to −0.4)	0.005	34.7%	34.3%	0.913
Heart failure	−1.6 (−2.8 to −0.5)	0.006	−1.2 (−2.5 to 0.1)	0.065	36.1%	34.3%	0.741
Chronic kidney disease	−0.1 (−0.7 to 0.4)	0.596	−0.1 (−0.6 to 0.5)	0.850	38.3%	34.4%	0.261
Retinopathy	0.1 (−0.5 to 0.7)	0.804	0.3 (−0.4 to 0.9)	0.429	29.4%	34.7%	0.126
Neuropathy	−0.9 (−1.4 to −0.4)	<0.001	−1.0 (−1.4 to −0.5)	<0.001	37.1%	34.4%	0.313
Erectile dysfunction	0.4 (−0.5 to 1.2)	0.401	0.7 (−0.2 to 1.5)	0.130	37.3%	34.5%	0.584

Note

Baseline PCS included in all models and all complications entered as time‐dependent covariates.

^a^ Each complication modelled separately.

^b^ All interval complications entered in the same model.

**TABLE 4:** Association of interval complications with mental quality of life

	Model Estimates				% with ≥2.5‐Point reduction		
	Unadjusted^a^	*p*‐value	Adjusted^b^	*p*‐value	With Complication	Without Complication	*p*‐value
Coronary artery disease	−0.8 (−1.7 to 0.0)	0.055	−0.8 (−1.7 to 0.2)	0.106	43.7%	38.3%	0.112
Cerebrovascular disease	−2.6 (−4.9 to −0.3)	0.030	−2.7 (−5.3 to −0.1)	0.045	55.3%	38.4%	0.032
Peripheral artery disease	−0.5 (−1.7 to 0.6)	0.369	−0.5 (−1.7 to 0.8)	0.457	42.4%	38.4%	0.378
Heart failure	−1.1 (−2.6 to 0.4)	0.163	−1.6 (−3.3 to 0.1)	0.059	44.4%	38.4%	0.294
Chronic kidney disease	0.4 (−0.3 to 1.1)	0.223	0.6 (−0.1 to 1.2)	0.116	39.3%	38.9%	0.908
Retinopathy	1.4 (0.6 to 2.2)	0.001	1.5 (0.7 to 2.3)	<0.001	33.0%	39.1%	0.083
Neuropathy	−0.5 (1.1 to 0.1)	0.121	−0.5 (−1.2 to 0.1)	0.095	42.2%	38.7%	0.197
Erectile dysfunction	0.5 (−0.6 to 1.6)	0.353	0.7 (−0.5 to 1.8)	0.252	42.2%	38.8%	0.537

Note

Baseline MCS included in all models and all complications entered as time‐dependent covariates.

^a^ Each complication modelled separately.

^b^ All interval complications entered in the same model.

In the repeated measures models that examined each interval complication in isolation, a new diagnosis of coronary artery disease, peripheral artery disease, heart failure and neuropathy was each associated with a small decrement in SF‐36 PCS score (Table 3) whereas a new cerebrovascular event was associated with a decrement in SF‐36 MCS score and a new retinopathy was associated with a better SF‐36 MCS score (Table 4). When all interval complications were considered in the same model, to account for different complications occurring in the same patient, results were similar, although the associations of interval coronary disease and heart failure with worse SF‐36 scores were no longer statistically significant (point estimates were similar).

## 4 DISCUSSION

In a prospective global cohort of patients with T2D initiating second‐line glucose‐lowering therapy, several T2D‐related complications were associated with a reduction in quality of life over time. Incident peripheral artery disease and neuropathy were associated with worse physical quality of life whereas incident cerebrovascular disease was associated with worse mental quality of life. This latter finding was most prominent, with over half of patients who had an interval cerebrovascular event reporting a clinically meaningful decline in SF‐36 MCS scores. Our data highlight the need to improve the prevention, early detection and management of vascular complications to maintain quality of life in individuals with T2D.

### 4.1 Prior studies

There are few studies that have examined the impact of complications of T2D with quality of life. A few cross‐sectional studies suggested that people with complications had worse quality of life compared with those without complications.^9^, ^10^, ^11^, ^12^, ^13^ In a cohort of US veterans with diabetes, complications that impacted functional capacity—peripheral neuropathy and peripheral artery disease—adversely affected quality of life, although these complications could not fully explain the impairments observed in the overall cohort where 87% reported poor physical functioning and 86% reported poor health perceptions.^13^ Importantly, patients with complications are different both demographically and clinically compared with those without complications. This markedly increases risk of confounding with cross‐sectional comparisons, which was evident in comparison of baseline SF‐36 data among those with or without complications. In our study, as each patient functioned as his or her own control, we were able to isolate the immediate effect of the complication on physical and mental quality of life, independent of patient factors.

### 4.2 Implications

Each of the complications of T2D can adversely impact quality of life independent of diabetes. Macrovascular complications can cause symptoms such as angina,^18^ claudication,^19^ dyspnoea^20^ or weakness^21^ that can markedly impair patients’ functional capacity and their ability to maintain social networks, increasing risk for depression and worsening quality of life. The impact of microvascular complications on quality of life is less precise. Retinopathy and nephropathy in their early stages are unlikely to cause symptoms or limitations that would adversely impact quality of life, but at the later stages of blindness and end‐stage renal disease, would be expected to have a marked negative impact on patients.^22^, ^23^ In contrast, neuropathy and erectile dysfunction are typically not diagnosed until there are symptomatic manifestations, which likely explains the association of neuropathy with worse physical quality of life, although we had hypothesized that erectile dysfunction would be associated with a similar decrement in mental quality of life,^24^ which was not seen in our study. Preventing complications is already an important goal in the treatment of T2D, as they increase the risk for additional complications, hospitalizations and death.^5^, ^25^ Our study enhances our understanding of the implications of these complications on peoples’ lives, including identifying which complications have a more immediate negative impact. Furthermore, these data could be used to inform models to estimate the potential benefit or cost‐effectiveness of interventions for T2D that reduce the risk of complications.

### 4.3 Limitations

First, it is important to note that DISCOVER is an observational study designed to describe processes of care in real‐world clinical practice and so screening for complications was not mandatory and the presence and severity of complications were not assessed using standard operating procedures and were not adjudicated. Second, although DISCOVER is a 3‐year study, it is possible that some complications impact quality of life in the longer term, particularly as the severity of the complications increases. This is particularly notable for microvascular complications, where a new diagnosis (eg chronic kidney disease) may not markedly impact quality of life but a more advanced form of the condition (eg end‐stage renal disease) would. Third, we were unable to account for other interval events, such as injuries or other deteriorations in health unrelated to T2D, which could have had a negative impact on quality of life over time. Fourth, although the analytic cohort was both large and diverse (30 countries across 6 continents), it is unknown if these results apply to patients in other countries and healthcare systems are not known. Even within DISCOVER, we had to exclude some countries and patients due to missing quality of life or complication data, further challenging the generalizability. Finally, the SF‐36 is a generic health‐related quality of life measure and therefore less sensitive to change. Complications of T2D may impact quality of life in ways that are not captured by the SF‐36 (eg chest pain, shortness of breath).

## 5 CONCLUSION

In a prospective global study of patients with T2D, we found that macrovascular complications and neuropathy are associated with a negative impact on quality of life. Vascular complications of T2D are known to adversely impact survival. Our results highlight the negative impact of these complications on quality of life—another important aspect of the overall health and well‐being of patients with T2D with its preservation being a key goal of management. While our estimates were relatively small, it is important to note these are the early effects of the complications on quality of life, which could increase over time as the severity of the complications progress. Our results provide further support to prevent, detect and appropriately manage vascular complications of T2D.

## CONFLICT OF INTEREST

SVA and FT: Employees of Saint Luke's Mid America Heart Institute, which received funding from AstraZeneca. KK: Investigator‐initiated studies for Astra Zeneca, Novartis, Novo Nordisk, Sanofi‐Aventis, Lilly and Merck Sharp & Dohme, Boehringer Ingelheim, Bayer, Berlin‐Chemie AG/Menarini Group, Janssen and Napp. HC, PF, JM and AC: Employees of AstraZeneca. AN: Receive support from AstraZeneca to attend DISCOVER planning and update meetings; honoraria from AstraZeneca, Eli Lilly, Medtronic and Novo Nordisk; research support from Artsana, Dexcom, Novo Nordisk and Sanofi. MBG: Receive support from AstraZeneca to attend DISCOVER planning and update meetings; honoraria from Merck‐Serono. LJ: Receive support from AstraZeneca to attend DISCOVER planning and update meetings; honoraria from AstraZeneca, Bayer, Boehringer Ingelheim, Bristol‐Myers Squibb, Eli Lilly, Merck Sharp & Dohme, Novartis, Novo Nordisk, Takeda, Sanofi and Roche, and research support from AstraZeneca, Bristol‐Myers Squibb, Eli Lilly, Merck Sharp & Dohme, Novartis, Roche and Sanofi. MVS: Receive support from AstraZeneca to attend DISCOVER planning and update meetings; honoraria from AstraZeneca, Boehringer Ingelheim, Eli Lilly, Merck Sharpe & Dohme, Novo Nordisk, Sanofi and Servier; research support from Novo Nordisk, Sanofi and Servier. HW: Receive support from AstraZeneca to attend DISCOVER planning and update meetings; honoraria from Astellas Pharma, AstraZeneca, Boehringer Ingelheim, Daiichi Sankyo, Sumitomo Dainippon Pharma, Eli Lilly, Kissei Pharmaceutical, Kowa Pharmaceuticals America Inc., Kyowa Hakko Kirin, Merck Sharp & Dohme, Mitsubishi Tanabe Pharma, Novartis, Novo Nordisk, Ono Pharmaceutical, Sanofi, Sanwa Kagaku Kenkyusho and Takeda; research support from Abbott, Astellas Pharma, AstraZeneca, Bayer, Benefit One Health Care, Boehringer Ingelheim, Bristol‐Myers Squibb, Daiichi Sankyo, Dainippon Sumitomo Pharma, Eli Lilly, Johnson & Johnson, Kissei Pharmaceutical, Kowa Pharmaceuticals America Inc., Kyowa Hakko Kirin, Merck Sharp & Dohme, Mitsubishi Tanabe Pharma, Mochida Pharmaceutical, Nitto Boseki, Novartis, Novo Nordisk, Ono Pharmaceutical, Pfizer, Sanofi, Sanwa Kagaku Kenkyusho, Taisho Toyama Pharmaceutical, Takeda and Terumo Corp. NH: former employee of AstraZeneca; owns company shares in AstraZeneca; consulting for Swedish Orphan Biovitrum. MK: Receive support from AstraZeneca to attend DISCOVER planning and update meetings; honoraria from Amgen, Applied Therapeutics AstraZeneca, Bayer, Boehringer Ingelheim, Eli Lilly, Janssen, Merck (Diabetes), Novo Nordisk, Sanofi and Vifor Pharma.

## AUTHOR CONTRIBUTION

**Suzanne V Arnold:** Conceptualization (lead); Formal analysis (supporting); Investigation (lead); Methodology (lead); Writing – original draft (lead); Writing – review & editing (lead). **Kamlesh Khunti:** Conceptualization (supporting); Data curation (supporting); Investigation (supporting); Writing – review & editing (supporting). **Fengming Tang:** Formal analysis (lead); Methodology (supporting); Writing – original draft (supporting); Writing – review & editing (supporting). **Hungta Chen:** Data curation (supporting); Investigation (supporting); Methodology (supporting); Resources (supporting); Supervision (supporting); Writing – review & editing (supporting). **A. Nicolucci:** Data curation (supporting); Investigation (supporting); Writing – review & editing (supporting). **Marilia B Gomes:** Data curation (supporting); Investigation (supporting); Writing – review & editing (supporting). **Li‐Nong Ji:** Data curation (supporting); Investigation (supporting); Writing – review & editing (supporting). **Marina V Shestakova:** Data curation (supporting); Investigation (supporting); Writing – review & editing (supporting). **Hirotaka Watada:** Data curation (supporting); Investigation (supporting); Writing – review & editing (supporting). **Andrew Cooper:** Data curation (supporting); Investigation (supporting); Resources (supporting); Writing – review & editing (supporting). **peter fenici:** Data curation (supporting); Investigation (supporting); Resources (supporting); Writing – review & editing (supporting). **Niklas Hammar:** Data curation (supporting); Investigation (supporting); Resources (supporting); Writing – review & editing (supporting). **Jesús Medina:** Data curation (supporting); Investigation (supporting); Resources (supporting); Writing – review & editing (supporting). **Mikhail Kosiborod:** Conceptualization (supporting); Investigation (supporting); Methodology (supporting); Resources (supporting); Supervision (lead); Writing – review & editing (supporting).

## Supporting information

Supplementary Material
