# Supplementary material for: Impact of micro‐ and macrovascular complications of type 2 diabetes on quality of life: Insights from the DISCOVER prospective cohort study
Source: Endocrinol Diabetes Metab. 2022 Jan 14;5(2):e00321. doi: 10.1002/edm2.321 (PMC8917866; doi:10.1002/edm2.321)
Supplement: Supplementary file 1 — Supplementary Material [file EDM2-5-e00321-s001.docx]

| **Supplemental Table 1. Countries in DISCOVER** | |
| --- | --- |
| **Africa** | **Europe** |
| Algeria | Austria |
| Egypt | Czechia |
| South Africa | France |
| Tunisia | Italy |
| Argentina | Netherlands |
| **Americas** | Poland |
| Brazil | Russia |
| Colombia | Spain |
| Costa Rica | Sweden |
| Mexico | **Mediterranean/Middle East** |
| Panama | Jordan |
| **Asia/Western Pacific** | Lebanon |
| Australia | Saudi Arabia |
| India | Turkey |
| Indonesia | UAE |
| Malaysia |  |
| South Korea |  |
| Taiwan |  |
| **Part of DISCOVER study but excluded from current analysis** | |
| Bahrain | Japan |
| Canada | Kuwait |
| China | Norway |
| Denmark | Oman |

| **Supplemental Table 2. Patient characteristics of those eligible but missing data versus analytic cohort** | | | |
| --- | --- | --- | --- |
|  | **Missing Data n=4606** | **Analytic Cohort n=7830** | **Standardized Differences** |
| Age, years | 57.0 ± 11.8 | 56.6 ± 11.6 | 3.4% |
| Female sex | 2163 (47.0%) | 3726 (47.6%) | 1.2% |
| Tobacco smoking |  |  |  |
| Non-smoker | 3141 (70.2%) | 5830 (75.8%) | 12.6% |
| Former smoker | 752 (16.8%) | 1029 (13.4%) | 9.5% |
| Current smoker | 580 (13.0%) | 828 (10.8%) | 6.8% |
| Body mass index, kg/m^2^ | 30.5 ± 6.2 | 29.6 ± 5.7 | 15.1% |
| Duration of diabetes, years | 6.0 ± 5.3 | 5.6 ± 5.1 | 7.7% |
| Systolic blood pressure, mmHg | 134.0 ± 17.3 | 131.4 ± 16.0 | 15.6% |
| Diastolic blood pressure, mmHg | 80.2 ± 9.8 | 79.9 ± 9.3 | 3.1% |
| HbA1c, % | 8.4 ± 1.8 | 8.4 ± 1.7 | 0.0% |
| Total cholesterol, mg/dL | 187.8 ± 49.0 | 185.3 ± 47.9 | 5.2% |
| LDL-cholesterol, mg/dL | 110.0 ± 39.9 | 107.7 ± 40.0 | 5.8% |
| Triglycerides, mg/dL | 182.1 ± 124.5 | 182.3 ± 123.4 | 0.2% |
| HDL-cholesterol, mg/dL | 45.4 ± 14.1 | 44.7 ± 12.9 | 5.2% |
| Creatinine, mg/dL | 1.0 ± 1.1 | 1.0 ± 1.0 | 0.0% |
| ACE-I or ARB | 1798 (39.0%) | 3090 (39.5%) | 1.0% |
| Beta blocker | 795 (17.3%) | 1223 (15.6%) | 4.6% |
| Statin | 2171 (47.1%) | 3492 (44.6%) | 5.0% |
| Aspirin | 922 (20.0%) | 1339 (17.1%) | 7.5% |
| Microvascular complications (baseline) | 893 (19.5%) | 1422 (18.2%) | 3.3% |
| Chronic kidney disease | 382 (8.3%) | 559 (7.1%) | 4.5% |
| Retinopathy | 182 (4.0%) | 238 (3.0%) | 5.4% |
| Neuropathy | 355 (7.7%) | 729 (9.3%) | 5.7% |
| Erectile dysfunction | 157 (3.4%) | 208 (2.7%) | 4.1% |
| Macrovascular complication (baseline) | 677 (14.8%) | 966 (12.3%) | 7.3% |
| Heart failure | 171 (3.8%) | 314 (4.0%) | 1.0% |
| Coronary artery disease | 452 (9.9%) | 677 (8.6%) | 4.5% |
| Cerebrovascular disease | 144 (3.2%) | 159 (2.0%) | 7.5% |
| Peripheral artery disease | 107 (2.3%) | 122 (1.6%) | 5.1% |

LDL, low density lipoprotein; HDL, high density lipoprotein; ACE-I, angiotensin converting enzyme-inhibitor; ARB, angiotensin II receptor blocker
